# Wrong, but useful: regional species distribution models may not be improved by range‐wide data under biased sampling

**DOI:** 10.1002/ece3.3834

**Published:** 2018-01-24

**Authors:** Ahmed El‐Gabbas, Carsten F. Dormann

**Affiliations:** ^1^ Department of Biometry and Environmental System Analysis University of Freiburg Freiburg Germany

**Keywords:** elastic net, Maxent, point‐process model, presence‐only data, regional data, sampling bias, species distribution modeling

## Abstract

Species distribution modeling (SDM) is an essential method in ecology and conservation. SDMs are often calibrated within one country's borders, typically along a limited environmental gradient with biased and incomplete data, making the quality of these models questionable. In this study, we evaluated how adequate are national presence‐only data for calibrating regional SDMs. We trained SDMs for Egyptian bat species at two different scales: only within Egypt and at a species‐specific global extent. We used two modeling algorithms: Maxent and elastic net, both under the point‐process modeling framework. For each modeling algorithm, we measured the congruence of the predictions of global and regional models for Egypt, assuming that the lower the congruence, the lower the appropriateness of the Egyptian dataset to describe the species' niche. We inspected the effect of incorporating predictions from global models as additional predictor (“prior”) to regional models, and quantified the improvement in terms of AUC and the congruence between regional models run with and without priors. Moreover, we analyzed predictive performance improvements after correction for sampling bias at both scales. On average, predictions from global and regional models in Egypt only weakly concur. Collectively, the use of priors did not lead to much improvement: similar AUC and high congruence between regional models calibrated with and without priors. Correction for sampling bias led to higher model performance, whatever prior used, making the use of priors less pronounced. Under biased and incomplete sampling, the use of global bats data did not improve regional model performance. Without enough bias‐free regional data, we cannot objectively identify the actual improvement of regional models after incorporating information from the global niche. However, we still believe in great potential for global model predictions to guide future surveys and improve regional sampling in data‐poor regions.

## 1 INTRODUCTION

Species distribution models (SDMs) are statistical methods that relate species information (either presence‐only or presence–absence) to environmental variables to infer spatially explicit habitat suitability. They are being used intensively as a standard tool for estimating potential range shifts under climate change, assessing invasion risk, locate future survey sites, and conservation planning and prioritization (Araújo, Alagador, Cabeza, Nogués‐Bravo, & Thuiller, 2011; Guisan & Zimmermann, 2000; Guisan et al., 2013; Rodríguez, Brotons, Bustamante, & Seoane, 2007; Thuiller et al., 2005). Although these methods have limitations and uncertainties (Araújo & Guisan, 2006; Dormann, Purschke, Márquez, Lautenbach, & Schröder, 2008; Guisan & Thuiller, 2005), they constitute the best available tools when not much detailed information on the ecology and physiology of the species is available (Warren, Wright, Seifert, Shaffer, & Franklin, 2014).

In developing countries, the majority of species sightings are scattered, opportunistic, and recorded mainly in museum catalogues, personal collections, and the literature. Due to political instability and limited funds dedicated to wildlife conservation (amongst other reasons), there is no systematic nation‐wide sampling scheme for collecting biological information in most developing countries. Many of these countries do not share their biodiversity data, making them highly under‐represented at international data depositories, such as the Global Biodiversity Information Facility (GBIF), with many more records from countries with high GDP (Newbold, 2010). Furthermore, data from developing countries are particularly (but not exclusively) spatially biased (more records from accessible locations near roads and cities) and taxonomically biased (toward larger or charismatic species). Spatial bias poses a problem for SDMs, which, in their default approach, assume that available presence locations represent a random (representative) sample in the environmental/geographical space, with no spatial dependencies (Elith et al., 2011; Renner et al., 2015). This assumption is hardly ever met due to sampling bias, imperfect detectability and spatial auto‐correlation (Guillera‐Arroita et al., 2015). When high sampling bias exists, SDM predictions provide an estimate not necessarily of the species suitability, but more of the patterns of the sampling effort and detectability (Elith et al., 2011; Yackulic et al., 2013). Several methods have been proposed to correct for sampling bias (e.g., target‐group background: Phillips et al., 2009; spatial filtering: Anderson & Raza, 2010; sampling bias predictors: Warton, Renner, & Ramp, 2013); however, no method seems to be able to fully correct for sampling bias in presence‐only data (El‐Gabbas & Dormann, 2017; Merow et al., 2014).

One of the major challenges of SDM studies is how to determine the extent of the study area appropriately. Study area should be objectively determined to cover accessible areas by the species within its known complete range, allowing for wider range of environmental variation and extremes occupied by the species (Barve et al., 2011; Raes, 2012; Sánchez‐Fernández, Lobo, & Hernández‐Manrique, 2011). However, it is common that study areas are unjustifiably determined based on geographical or political borders for regional/local conservation actions, resulting in models calibrated with a limited range of environmental conditions that do not capture much of the species' niche and hence is insufficient to describe its environmental tolerance (Raes, 2012; Titeux et al., 2017). This leads to the truncation of the estimated response curves, underrepresentation of areas of suitable habitats, and limiting the predictive power of the models (Sánchez‐Fernández et al., 2011; Thuiller, Brotons, Araújo, & Lavorel, 2004). This is more problematic when the aim of the study is to extrapolate beyond the training range, either in time or space (Barbet‐Massin, Thuiller, & Jiguet, 2010; Thuiller et al., 2004), or in situations where available data are few, opportunistic, or with high (typically unknown) sampling bias. The paucity of available records in developing countries, coupled with clear signs of sampling bias and limited local environmental gradients, makes it challenging to establish robust SDMs for a variety of taxonomic groups at the national scale.

**Figure 1:**
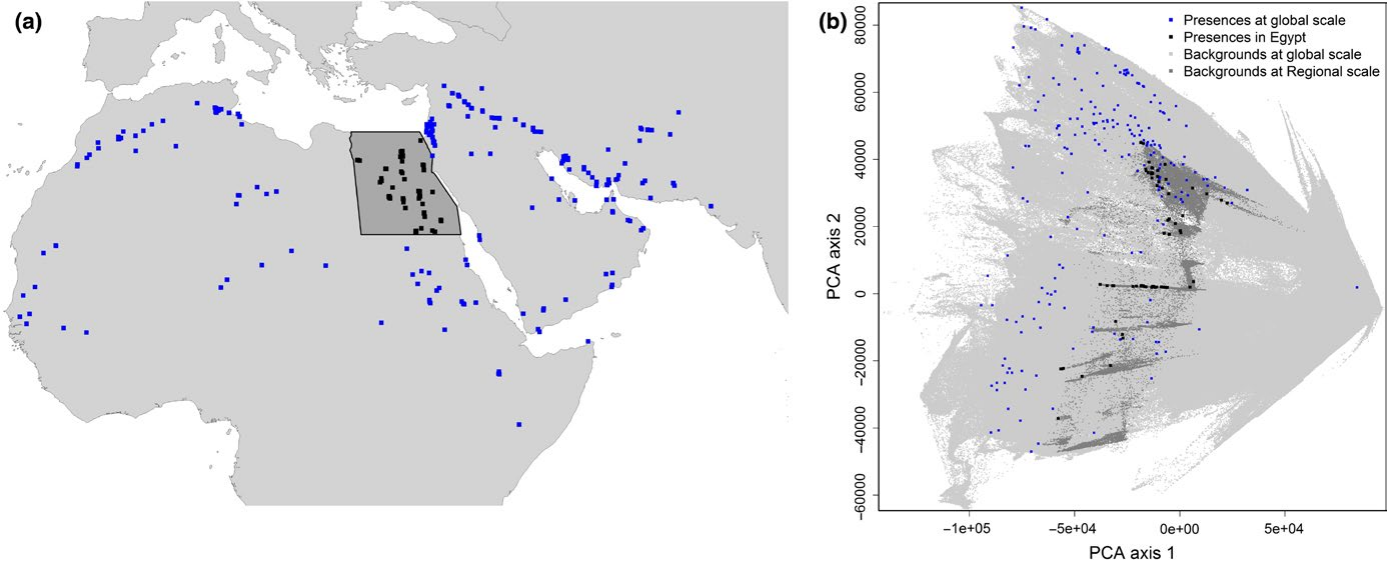
The distribution of *Asellia tridens* at spatial (a) and environmental (b) space. The map a shows the species‐specific global extent of this species, with dots representing the spatial distribution at global (blue) and regional (black) scales. Panel b shows a scatterplot of the first two PCA axes of all available environmental covariates within the entire study area. The first two axes account for 94.2% of the environmental variation. Blue and black dots are presence locations of the species outside and inside Egypt, respectively; light gray points are pixels without any sightings at global scale; dark gray points represent the available environmental space in Egypt. Figure S1 shows equivalent plot for all study species together

In this article, we evaluate the adequacy of regional presence‐only data (in this case from within a developing country's political borders) for constructing SDMs. More specifically, we compare bat occurrence predictions from regional and global SDMs for the country of Egypt, in many respects exemplary for developing countries. Egypt shows much lower environmental variability compared to the global extents of the species (see Figures 1 and S1) and comprises only a small proportion of available global records. This makes the quality of regional SDMs, that is, those built only on the sparse Egyptian data, questionable. Global models (at species‐specific global range) should in this case be more reliable than regional models (in Egypt) in describing the climatic niche of species because they are calibrated with a much higher number of presences and capture a much wider range of occupied (or, more generally, accessible) environmental conditions (Pearson, Dawson, & Liu, 2004). Thus, we evaluate predictions from regional and global SDMs for Egypt, arguing that the less similar they are, the more the local data describe sampling effort rather than the ecology of bats. Furthermore, we investigate how much correction for sampling bias (using bias predictors, in both regional and global SDMs) helps to improve the local predictions for Egypt.

Predictions from global models interpolated to Egypt represent a spatial‐explicit information on the species potential distribution that is independent from regional data available from Egypt, and thus can be useful to improve predictions of regional models when used as additional predictors (cf. “informative offset”: Merow, Allen, Aiello‐Lammens, & Silander, 2016). We explore how much global predictions (interpolated to Egypt) improve Egyptian regional models when used as predictor “prior” to describe the environmental niche (again, with and without correcting for sampling bias).

## 2 METHODS

### 2.1 Study design and species

This study builds on a comparison of methods to correct for sampling biases (El‐Gabbas & Dormann, 2017), adding an evaluation of regional species distribution models based on national records. We collected records for Egyptian bat species (from within Egypt and their global extents) from different sources (Appendix S1 and El‐Gabbas & Dormann, 2017). Four species with fewer than eight unique sightings in Egypt were excluded from the analyses, yielding a total of 17 species (Table S1). For the selected species, we created regional models using presence locations and environmental data only for Egypt (“regional SDMs”). “Regional” refers here to a geographic extent much smaller than the range of the species, but of much coarser grain than a local dataset. We also created analogous models across the global range (“global SDMs”): These models were made for each species‐specific global extent (a buffered bounding box around all global records), excluding Egyptian records to maintain independence (and to allow for valid comparisons) between the predictions of the regional and global models (see below; and El‐Gabbas & Dormann, 2017 for details). Both scales are nested in geographical and environmental space: Our regional models are calibrated within a subset of each species‐specific global extent. At either scale, we used two modeling algorithms under the point‐process modeling framework (Maxent and elastic net; Renner et al., 2015), with two options on dealing with sampling bias (with and without bias correction), and evaluated the results using spatial‐block cross‐validation (Roberts et al., 2017).

### 2.2 Environmental variables

Potential environmental predictors (at the total study area covering both scales) and species records were projected into Mollweide equal‐area projection at a resolution of 5 × 5 km^2^. Using the same pixel size and projection maintains consistency of the analyses between regional and global models (Budic, Didenko, & Dormann, 2016). As the correlation between predictors varies from one study area to another, different environmental predictor combinations were used at regional and global scales. Some predictors were not useful at the regional scale, and hence were excluded a priori; for example, precipitation of driest month does not show any variability across Egypt because most of Egypt receives no precipitation at all in summer, reflecting its hyper‐arid climate (El‐Gabbas, Baha El Din, Zalat, & Gilbert, 2016). We ensured minimum multi‐collinearity at both scales by selecting only predictors that maintain a maximum generalized variance inflation factor value less than 3 (see Table S2 for the list of predictors used at either scale).

### 2.3 Modeling algorithms

We used two modeling algorithms: Maxent and elastic net. Maxent (Phillips & Dudík, 2008; v3.3.3k) is a machine‐learning presence‐background SDM algorithm. It outperforms other presence‐only SDM algorithms, especially at smaller sample sizes (e.g., Wisz et al., 2008), due to its use of (some form of) lasso regularization. Elastic net (Friedman, Hastie, & Tibshiani, 2010) is an extension of GLMs that uses “lasso” and “ridge” regularization rather than AIC to select the most suitable model, and hence is similarly resistant to overfitting. We applied both algorithms under the point‐process modeling framework following recommendations of Renner et al. (2015), changing some of Maxent's default settings (e.g., to “noautofeature,” “noaddsamplestobackground,” and “noremoveduplicates”), and used the implementation of “down‐weighted Poisson regression” for elastic‐net models. For each calibrated model of either algorithm, we adjusted against unnecessary complexity (Merow et al., 2014) using five‐fold spatial‐block cross‐validation, estimating the best combination of Maxent's feature classes and regularization multiplier based on maximizing the mean testing AUC (Muscarella et al., 2014), and the optimum α (which describes the balance between ridge and lasso) for elastic net.

### 2.4 Adjusting for sampling bias

In addition to “environment‐only” models (without bias correction), we use two different methods of predicting from models that incorporate bias: “bias‐predictor” and “bias‐corrected.” In both methods, we use sampling bias predictors as our estimate of bias: three layers describing distances to main roads, cities, and protected areas (Warton et al., 2013). Bias‐predictor models use the bias layers simply as an extra set of predictors, and during prediction also their values change. Bias‐corrected models try to factor out the bias by setting the bias variables to zero (see Warton et al., 2013). The three options for sampling bias (none, predictor, and correction) were applied to regional and global models, with bias predictors nested for regional scale within the global scale.

### 2.5 Model evaluation and the use of spatial priors

We evaluated regional model performance using AUC as a threshold‐independent metric. Despite the criticism of the use of AUC to evaluate the performance of presence‐only SDMs (e.g., Lobo, Jiménez‐Valverde, & Real, 2008), our use of AUC for comparisons between models of the same species, predictors, and study area is valid (Anderson & Gonzalez, 2011; Wisz et al., 2008). We did not use AUC to quantify model performance (goodness of fit), but rather as a measure of the relative ranking of predictions at testing presence and background locations. We calculated AUC on five‐fold spatial‐block cross‐validation to maintain spatial independence between training and testing data (Fithian, Elith, Hastie, Keith, & O'Hara, 2015; Roberts et al., 2017): The same blocking structure (how spatial blocks are distributed into cross‐validation folds) is used for each species, with balanced prevalence among blocks and same block sizes, allowing for valid AUC comparisons for the same species. The mean value of testing AUC on cross‐validation is reported.

To quantify the efficacy of Egyptian data to construct SDMs, we calculated the geographical congruence (Schoener's D; Schoener, 1968; Warren, Glor, & Turelli, 2010) between continuous predictions of the global and regional SDMs for Egypt (scaled to sum to one; without and with bias correction). Our assumption is that the higher the geographical congruence, the more suitable the Egyptian records are to parameterize regional models. When assessing the congruence between maps we used all three bias options, while for regional comparisons based on AUC we only used the first two models (environment‐only and bias‐predictor), due to the lack of bias‐free testing‐data from Egypt required to evaluate bias‐corrected predictions. Geographical congruence and AUC gave similar results, indicating that geographical congruence indeed measured how similarly well, not how similarly poorly models predicted.

We then measured the improvement of regional SDMs after incorporating a spatial‐explicit information on the global climatic niche. More specifically, for each species we used predictions from the global SDM interpolated to Egypt (i.e., not using the Egyptian data, and thus referred to hereafter as “prior”) as an additional predictor to create a new set of regional models. We had three types of priors representing the predictions of global models for Egypt: 1) from the environment‐only model, “Prior_env‐only_”; 2) a prediction incorporating the bias layer as a predictor to adjust for sampling bias, “Prior_bias‐predicted_”; and 3) a prediction from a model that has factored out bias, “Prior_bias‐corrected_”. Modeling algorithms were not mixed, that is, global models from Maxent were used only for regional models with Maxent, and analogously for elastic‐net models. We quantified the improvement due to priors in two ways. First, we measured changes in model performance (AUC). Secondly, we calculated the map congruence between regional models' predictions in Egypt with and without incorporating priors: the higher the map congruence, the lower the contribution of the prior to the regional SDM. One‐tailed paired *t*‐test (*df* = 16) was used for comparisons between each pair of modeling algorithms, sampling bias options, and changes in AUC and map congruence.

## 3 RESULTS

**Figure 2:**
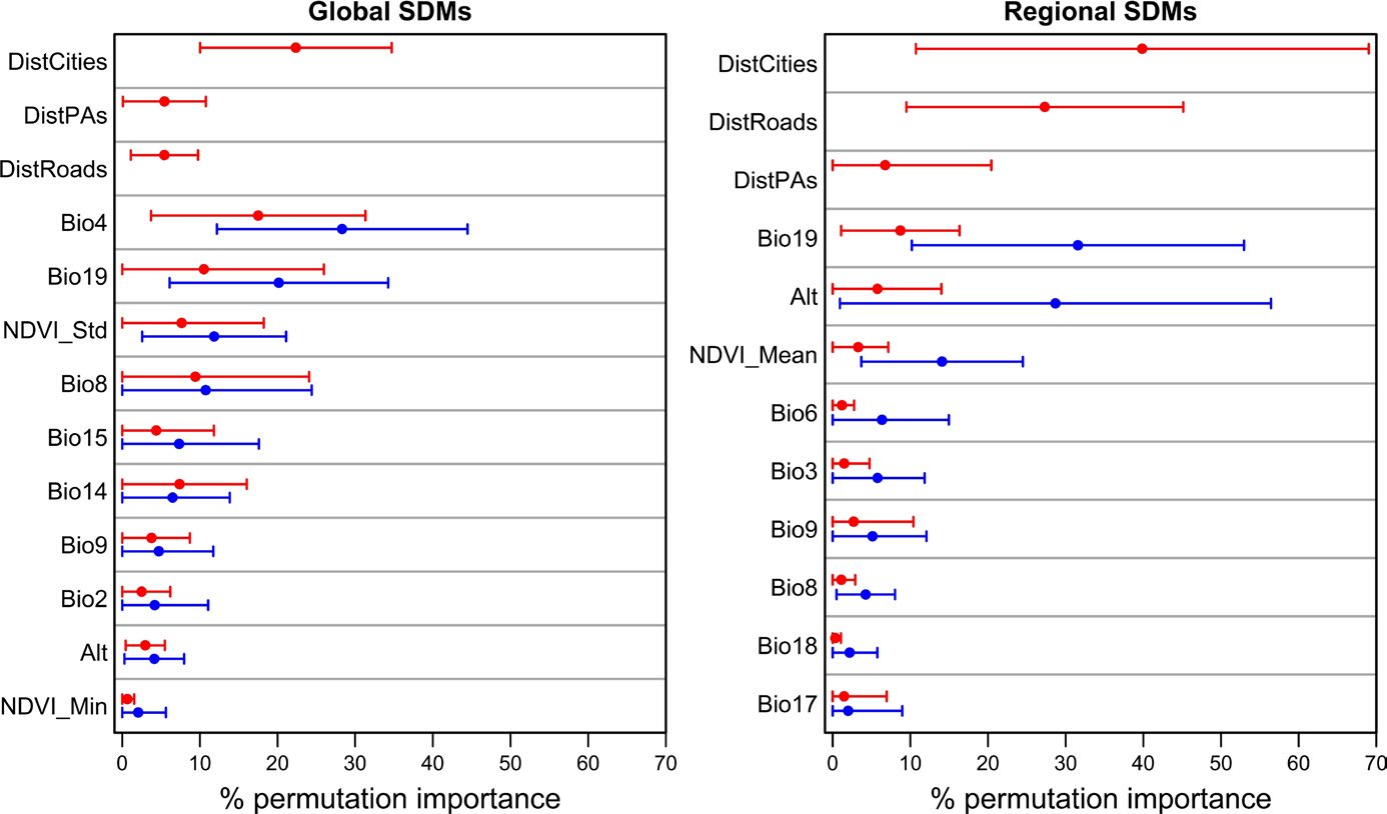
Mean permutation importance of environmental variables used at global (left) and regional (right) models (from Maxent). Dots and error bars represent the overall mean and standard deviation of the average permutation importance of the seventeen study species, respectively. Blue dots/bars represent environment‐only models; red dots/bars represent comparable models with accessibility bias variables incorporated as predictors. When included, bias predictors have a high contribution (particularly distance to main cities at both scales, and distance to roads in Egypt), compared to many environmental variables. For more details on the environmental variables used, see Table S2

The relative importance of environmental variables (permutation importance calculated by Maxent) varied at global and regional scales. When incorporated, the accessibility bias predictors at both scales had high Maxent permutation importance (particularly, “distance to cities” was of significantly higher importance than all but one variable [*p* < .05; nonsignificant only for Bio4 at global scale and Bio6 at regional scale], and “distance to roads” which had a significantly higher average importance than three different environmental variables at either scales; Figure 2). Furthermore, the response of species to environmental predictors was, unsurprisingly, different at both scales. For example, for *Eptesicus bottae* at the global scale, the response to precipitation of the coldest quarter increased sharply at low precipitation values (approx. 0–130 mm), then remained high or decayed depending on whether the global bias predictors were used or not, respectively (Figure S2a). At the regional scale, however, the species response was highest at extremely low precipitation values (around 10 mm), then declined sharply (Figure S2c).

### 3.1 Global versus regional SDMs

**Figure 3:**
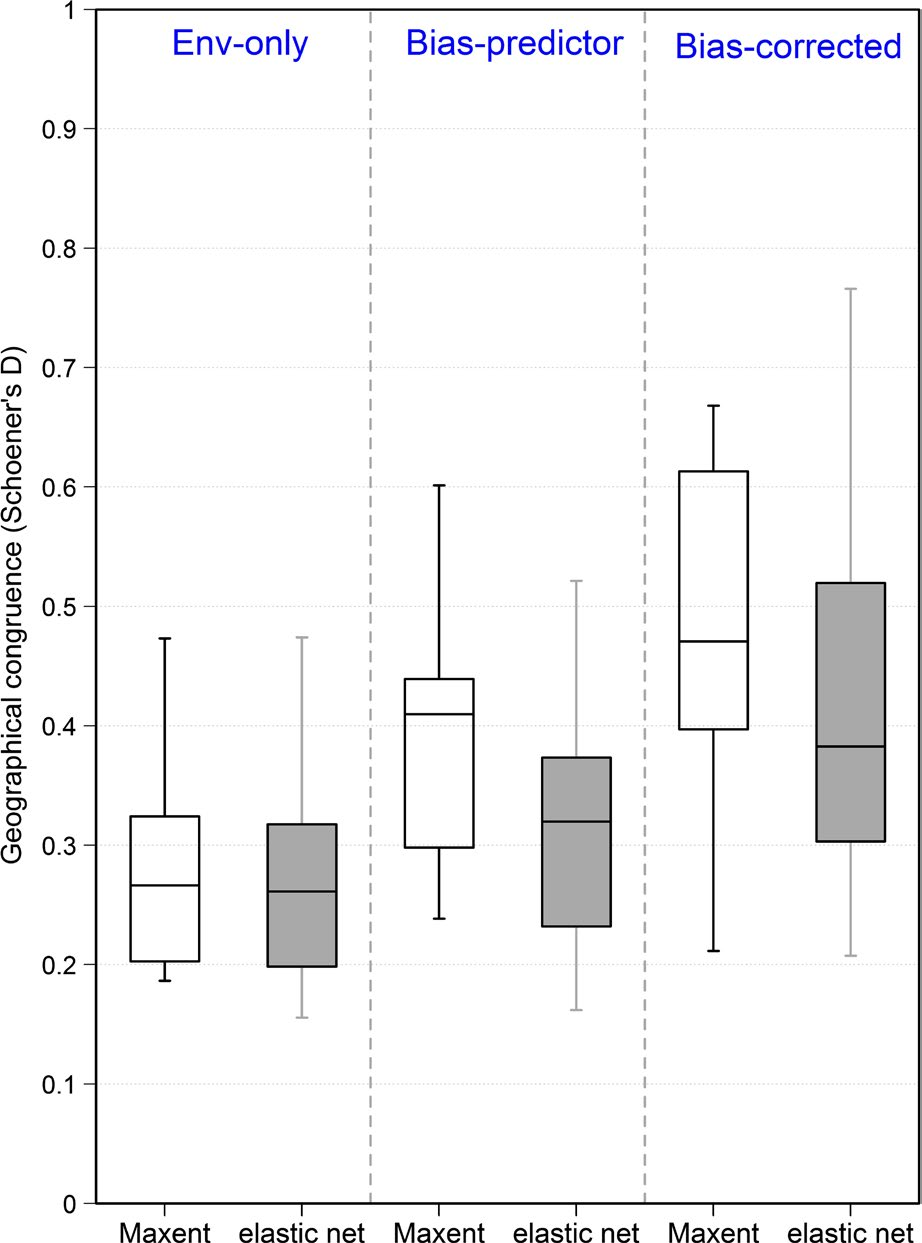
Boxplots for the geographical congruence (Schoener's *D*) between mean predictions of global and regional models for Egypt (with no priors). Schoener's *D* ranges from zero to one, representing situations of no to full congruence, respectively. “Env‐only” are models calibrated only with environmental variables. “Bias‐predictor” models add accessibility bias variables as predictors to the model. “Bias‐corrected” models also use bias variables to set bias to zero during prediction (i.e., bias factored‐out)

Different areas were identified as suitable in models either using data from the full range or just from Egypt, with low geographic congruence between the predictions of global and regional models for Egypt (Figure 3). The incorporation of bias predictors (at both scales) did not lead to substantial congruence improvement (yet statistically significant; all *p* < .01). The congruence was highest when bias‐corrected models were used (statistically higher than environment‐only and bias‐predicted models for Maxent and elastic net, *p* < .001). Maxent and elastic net yielded similar values for congruence, with an advantage of Maxent for bias‐predictor models (*p* < .05).

### 3.2 The use of prior information from the entire range

**Figure 4:**
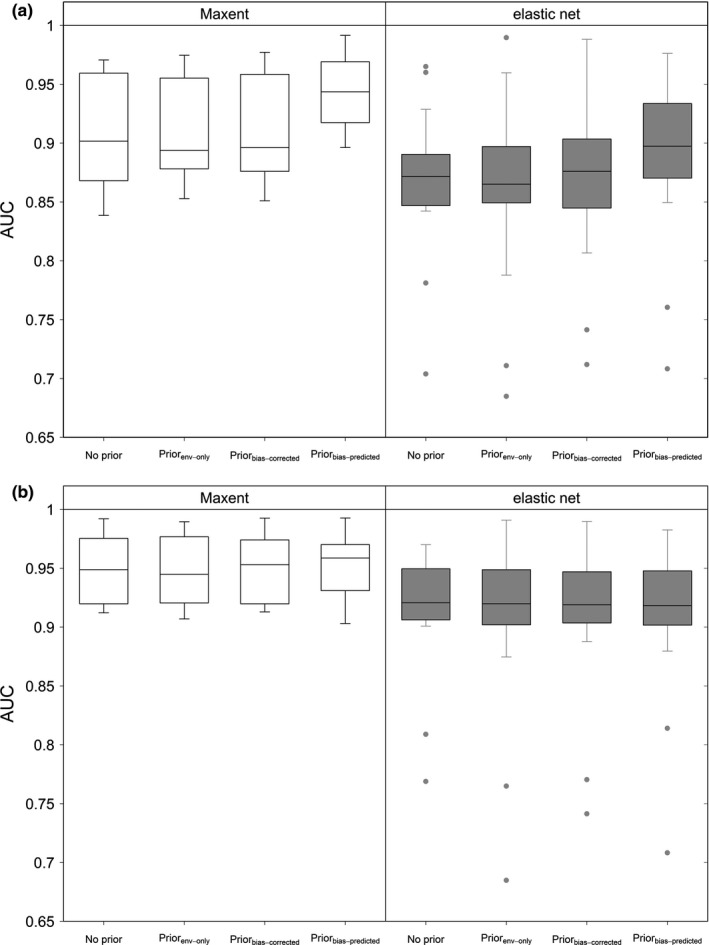
Boxplots for the mean AUC values (on cross‐validation) calculated for different options of modeling algorithms, bias manipulations, and priors. (a) A comparison between mean AUC values of no‐prior regional models and equivalent models that use different options of priors (without regional bias incorporated as predictors). (b) Same as a, with regional bias variables included as predictors

The use of priors did not lead to AUC improvement, except when using Prior_bias‐predictor_ (*p* < .05; Figure 4a). Results were similar for both Maxent and elastic net, with higher AUC values for Maxent (all *p* < .01). Maxent showed relatively low permutation importance of the different prior variables, except for Prior_bias‐predictor_ which had high contributions to the models (all *p* < .0001, although also with high variability; Figure S3, left panel).

**Figure 5:**
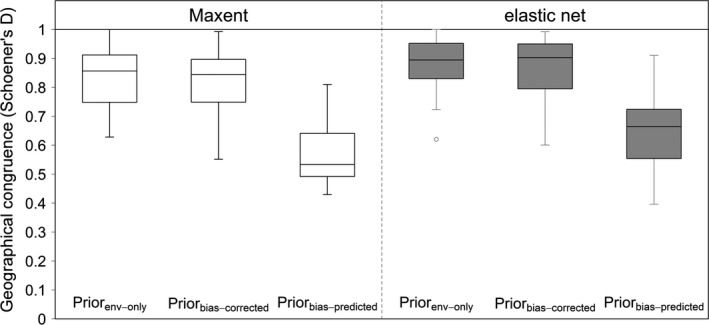
Geographical congruence between the predictions of regional SDMs calibrated without priors and the three versions of regional models that used a prior variable. Bias variables were not incorporated as predictors in the regional SDMs. There were three options of prior options: “Env‐only” are predictions of global SDMs without incorporating sampling bias; “Bias‐predictor” priors incorporate global accessibility bias variables as predictors in the model; and “Bias‐corrected” priors incorporate bias‐corrected (set to zero) predictions from global models for Egypt

The incorporation of prior variables as predictors yielded high geographical congruence between the predictions of regional models without and with priors (Figure 5). However, the congruence values depended on the prior used. The use of Prior_env‐only_ or Prior_bias‐corrected_ led to high congruence, indicating little additional information provided by the priors. In contrast, when Prior_bias‐predictor_ was used, geographical congruence was less pronounced (*p* < .001), suggesting that here information different from the regional data entered the model. Both Maxent and elastic net produced similar values for congruence, with slightly higher values for elastic net when Prior_bias‐predictor_ was used (marginally significant; *p* = .042).

### 3.3 Correction of regional sampling bias

When regional bias predictors were incorporated into the SDMs, the regional models performed better (higher AUC; all *p* < .05), leading to a negligible effect of priors (Figure 4b). Maxent has relatively higher AUC scores than elastic net (all *p* < .01). However, Prior_bias‐predictor_ showed equivalently high AUC values whether or not regional bias predictors were included (*p* > .7; see Figure 4a,b for a comparison). This was also evident by the much lower permutation importance of prior predictors when regional bias predictors were incorporated, with relatively higher importance for Prior_bias‐predictor_ (all *p* < .05; Figure S3, right panel).

Incorporating regional bias predictors led to similar patterns of congruence (between predictions of regional SDMs created with or without priors) to those which did not incorporate bias (Figure 5 vs. Figure S4, light gray boxes), with relatively lower congruence when Prior_bias‐predictor_ was used. However, bias‐correction (factoring out the bias) did not affect congruence for Maxent, while much lower congruence values were observed for elastic net whichever priors were used (Figure S4, dark gray boxes). In other words, regional bias correction led to less agreement between regional model predictions (with and without priors) for elastic net, regardless of which prior variables were used.

## 4 DISCUSSION

In this study, we evaluated how much improvement to the regional SDMs for Egypt occurs by incorporating additional information (the “priors”) representing the global climatic niche from outside Egypt. First, without providing information on regional bias (no regional bias correction), Prior_env‐only_ and Prior_bias‐corrected_ did not lead to improvements in the regional models: Similar AUC values (Figure 4a) and high geographical congruence (Figure 5) imply that they do not provide new information to the regional models. However, the use of Prior_bias‐predictor_ led on average to higher AUC and lower geographical congruence, signaling that new information was provided to the models. This was supported in Maxent models by the higher permutation importance of Prior_bias‐predictor_, compared to the other two options of priors (Figure S3, left panel). On the other hand, when regional bias predictors were incorporated, all models had improved AUC, whether or not priors were used (Figure 4b). Regional bias predictors describe the local bias existing in the Egyptian dataset, and their use led to higher AUC, in accordance with other studies (El‐Gabbas & Dormann, 2017; Warton et al., 2013). The use of regional bias predictors makes the contribution of priors negligible: Prior_env‐only_ and Prior_bias‐corrected_ had an extremely low contribution to these models, only slightly higher for Prior_bias‐predictor_ (Figure S3, right panel). Generally, Maxent and elastic net led to very similar results, with slightly higher discrimination ability for Maxent.

Prior_bias‐predictor_ implicitly contains information on the regional bias of the records in Egypt, because it represents predictions of equivalent global models calibrated with accessibility bias variables (regional bias variables represent a narrower range than their equivalent variables at global scale). In contrast to bias‐free predictions, the use of bias variables as predictors gives higher predicted suitabilities at locations of high accessibility (e.g., closer to roads and cities), which is the reason for high AUC scores when evaluation datasets are similarly biased (Warton et al., 2013). The available dataset for Egyptian bats is spatially‐biased, with more records collected near roads and cities (El‐Gabbas & Dormann, 2017), and hence Prior_bias‐predictor_ describes the available data better than the other two priors. The relatively modest contribution of Prior_bias‐predictor_, and even lower contribution of the other two priors, can be understood as the result of the unavailability of complete, bias‐free data from Egypt (see below). Furthermore, Prior_env‐only_ and Prior_bias‐corrected_ are highly correlated with some other environmental variables in Egypt (higher than for Prior_bias‐predictor_), particularly for Bio19 (precipitation of coldest quarter) and Bio9 (mean temperature of driest quarter; Figure S5), and hence to a large extent provide redundant information.

The three prior suitabilities show low geographical congruence with their corresponding regional predictions in Egypt (Figures 3 and S6, e.g., maps), meaning they (global models) identify different sites as suitable than do models based on Egyptian records. This can be explained by factors related to model misspecification (e.g., the variables used and violation of model assumptions), the difficulty of modeling widespread species with high accuracy (Stockwell & Peterson, 2002), the low quality of available data, or species‐specific reasons (e.g., species plasticity and the existence of ecotypes; Randin et al., 2006). We exclude environmental extrapolation as a reason for the on average low performance of the predictions of the global model for Egypt, as we included environmental data for the area of Egypt in these models (but not the records), and hence, the predictions are not outside the realm of the global model (and hence do not represent an extrapolation).

While it is advisable to check for collinearity at training and prediction scales (Elith, Kearney, & Phillips, 2010), it is not always easy to maintain a representative set of variables that are uncorrelated at both scales. Although we minimized the correlation between environmental variables at global and regional scales to avoid unnecessarily high variance in model parameters, the correlation among environmental variables is, inevitably, not constant over space (Dormann et al., 2013). Some of the variables used at the global scale have high correlation in Egypt, making the reliability of predictions in Egypt less stable (Dormann et al., 2013; Elith et al., 2010). Furthermore, the quality of environmental variables is not constant in space. For example, the WorldClim data (Hijmans, Cameron, Parra, Jones, & Jarvis, 2005; the source of most of the environmental variables used in this study) were adroitly prepared using interpolation of data from global weather stations. Weather stations are not evenly distributed in space: Climate data for areas such as Arabia and the Sahara (including Egypt) are interpolated using very few weather stations with high spatial clustering (see figure 1 in Hijmans et al., 2005), and hence, the interpolations are of potentially higher uncertainty that can affect the quality of calibrated models (Phillips, Anderson, & Schapire, 2006). This problem is not exclusive to the WorldClim data, but holds for any environmental layers derived from spatially‐biased weather stations.

The environmental variables used may have been insufficient to characterize the species niche (Phillips et al., 2006). It is recommended to use proximal predictors (e.g., food sources or suitable roosting sites for bats) that directly describe the required resources and physiological limits than more indirect distal predictors (e.g., altitude; Austin, 2007; Merow et al., 2014). The use of proximal variables increases the transferability of models in space (Elith & Leathwick, 2009; Franklin, 2009). However, determining a set of species‐specific proximal predictors is not possible without detailed knowledge of the ecology and physiology of each species, either unknown for most species (especially for bats) or not yet available at large scales (e.g., abundance of prey; Merow et al., 2014; Herkt, Matthias, Barnikel, Skidmore, & Fahr, 2016; Petitpierre, Broennimann, Kueffer, Daehler, & Guisan, 2017). The majority of SDM studies use (the easier to obtain) distal variables as surrogates for proximal variables; however, even if distal variables can indirectly describe the species requirements, the correlation between proximal and distal variables is not constant in space (Dormann et al., 2013; Elith & Leathwick, 2009; Merow et al., 2014). Examples of missed variables which can potentially improve model transferability for bats include locations of suitable roosting and foraging sites, proximity to water, food sources (Herkt et al., 2016; Razgour, Rebelo, Di Febbraro, & Russo, 2016). Regional models were calibrated for a limited environmental range (Figure S1), potentially contributing to the disagreement between regional and global model predictions.

While excessive model complexity can lead to overfitting to training data and consequent limited model transferability in space and time, we reject overfitting as a reason for the limited usefulness of priors. We limited overfitting using regularized modeling approaches, calibrated by spatial cross‐validation blocks in a way that balances the number of presence locations and environmental variability between cross‐validation folds (avoiding extrapolation) and adequately constrains the complexity of (both regional and global) models. That said, it is not clear how much model complexity optimization is affected by the limited number and quality of records (including sampling bias).

Predictions from global models interpolated to Egypt may well still describe the potential distribution of bats in Egypt. Their limited usefulness in our study only shows that the global dimension did not add new information, given the limitations of the available data from Egypt. If unbiased occurrence data were available, global models may indeed predict well in Egypt. Moreover, available bat records in Egypt are few and spatially‐biased toward easily accessible areas, with the majority collected from relatively old literature and museum specimens. Most are opportunistic data gathered with an unknown sampling strategy (see Appendix S1). Due to their nocturnal and elusive behaviour, high maneuverability, and the need for specialized bat detectors for effective recording, it is challenging to obtain high‐quality records for bats in developing countries (Razgour et al., 2016). Information on their geographical distribution is very limited, making bats highly under‐represented in SDM studies (Herkt et al., 2016; Razgour et al., 2016), and Egypt is no exception. Finally, sampling bias can strongly affect model quality (Phillips et al., 2009), and while we attempted to correct for sampling bias in our models, we cannot quantify the efficiency of bias correction without bias‐free data for comparison (Phillips et al., 2009; Warton et al., 2013), unavailable in most presence‐only studies, especially in developing countries. The results of this study call for improved, systematic sampling of species occurrences in regions where currently only biased and scarce data are available.

## 5 CONCLUSION

We have shown that the use of global bat data did not improve regional model performance for Egypt. We relate this to the difficulty of calibrating SDMs of widespread species at extremely large study areas that cover many biogeographical regions and to data quality issues (mainly the quantity of available data dominated by high sampling bias). Due to the lack of high‐quality data and limited environmental gradients in Egypt, regional SDMs seem to be insufficient to determine new survey sites (a point also made by Sánchez‐Fernández et al., 2011). Improving the sampling of fauna and flora species from data‐poor countries (such as Egypt, particularly from the less visited areas) would enhance regional SDMs in these countries and consolidate the usefulness of these models to discover new populations.

Although our results showed that predictions from global SDMs failed to improve regional predictions calibrated with low‐quality and spatially‐biased data, we still believe in great potential for SDMs that integrates global and regional data to improve future local sampling in data‐poor countries like Egypt. Patterns of potential distribution (of global models interpolated to Egypt) can guide future surveys and help to discover new populations. In our analyses, we excluded Egyptian data for creating the global models to maintain consistency of comparisons between predictions of regional and global models. However, this is not necessary for real applications, and it would seem preferable to include regional data in a comprehensive model that covers the biogeographical region to improve model predictability. For example, to improve sampling of under‐reported bat species in Egypt, we think that a larger‐scale model should be created, with the study area determined objectively based on the available data from Egypt and adjacent arid areas (e.g., Arabia and the Sahara) in order to meet the stationarity assumption (constant species–environment relationships with no change in niche characteristics; Anderson & Gonzalez, 2011; Dormann et al., 2012) and then crop the prediction maps to Egypt. This is of mutual benefit not only for Egypt, but also for targetting efforts in the adjacent areas as well, which can help to improve the conservation status of some species. However, obtaining enough data from adjacent areas will remain challenging for many species.

## AUTHOR CONTRIBUTIONS

AE‐G and CFD contributed to idea and design of study, and comments and revisions; AE‐G contributed to data curation and statistical analysis, and first drafted the writing. Both authors contributed critically to the drafts and gave final approval for publication.

## CONFLICT OF INTEREST

None declared.
