# Supplementary material for: Wrong, but useful: regional species distribution models may not be improved by range‐wide data under biased sampling
Source: Ecol Evol. 2018 Jan 24;8(4):2196–206. doi: 10.1002/ece3.3834 (PMC5817152; doi:10.1002/ece3.3834)
Supplement: Supplementary file 1 [file ECE3-8-2196-s001.pdf]

El-Gabbas A. & Dormann C. F.: *Wrong, but useful: regional species distribution models may not be improved by range-wide data under biased sampling.* Ecology & Evolution.

Supporting information

Appendix 1: List of literature resources used for extracting Egyptian bat records.

For the full list of literature used to extract records used in the global models, see Appendix 2 in El-Gabbas & Dormann (2017)

Please note that many records were cross-cited in many references, and such records were merged as one record to avoid spurious indication of predicted intensity when the same sighting is used many times.

ACR (2015) **African Chiroptera Report**. 2015. African Bats, Pretoria, i - xix, 1 - 7001 pp.

Ali R.A. (2011) **Molecular Phylogenetic Relationship Between and Within the Fruit Bat (*Rousettus aegyptiacus*) and the Lesser Tailed Bat (*Rhinopoma hardwickei*) Deduced From RAPD-PCR Analysis**. Journal of American Science, 7(10):678-687.

Andersen K. (1906) **On some new or little-known bats of the genus *Rhinolophus* in the collection of the Museo Civico, Genoa**. Annali del Musei Civico di Storia Naturale di Genova, (3a)(2):173-195.

Anderson J. (1902) **Zoology of Egypt: Mammalia (compiled by W.E. de Winton)**. London: Hugh Rees Ltd.

Benda P., Abi-Said M., Bartonička T., Bilgin R., Faizolahi K., Lučan R.K., Nicolaou H., Reiter A., Shohdi W.M., Uhrin M., Horáček I. (2011) ***Rousettus aegyptiacus* (Pteropodidae) in the Palaearctic: list of records and revision of the distribution range**. Vespertilio, 15: 3–36.

Benda P., Al-Juaid M.M., Reiter A., Nasher A.K. (2011) **Noteworthy records of bats from Yemen with description of a new species from Socotra**. Hystrix, n.s., 22(1):23–56.

Benda P., Andreas M., Kock D., Lučan R., Munclinger P., Nová P., Obuch J., Ochman K., Reiter A., Uhrin M., Weinfurtová D. (2006) **Bats (Mammalia: Chiroptera) of the Eastern Mediterranean. Part 4. Bat fauna of Syria: distribution, systematics, ecology**. Acta Societatis Zoologica Bohemicae, 70:1–329.

Benda P., Andriollo T., Ruedi M. (2014) **Systematic position and taxonomy of *Pipistrellus deserti* (Chiroptera: Vespertilionidae)**. Mammalia, 79(4):419-438.

Benda P., Dietz C., Andreas M., Hotový J., Lučan R., Maltby A., Meakin K., Truscott J., Vallo P. (2008) **Bats (Mammalia: Chiroptera) of the Eastern Mediterranean and Middle East. Part 6. Bats of Sinai (Egypt) with some taxonomic, ecological and echolocation data on that fauna**. Acta Societatis Zoologicae Bohemicae, 72(1-2):1-103.

Benda P., Faizolâhi K., Andreas M., Obuch J., Reiter A., Ševčík M., Uhrin M., Vallo P., Ashrafi S. (2012) **Bats (Mammalia: Chiroptera) of the Eastern Mediterranean and Middle East. Part 10. Bat fauna of Iran**. Acta Societatis Zoologicae Bohemicae, 76:163–582.

Benda P., Gvoždík V. (2010) **Taxonomy of the genus *Otonycteris* (Chiroptera: Vespertilionidae: Plecotini) as inferred from morphological and mtDNA data**. Acta Chiropterologica, 12(1):83-102.

Benda P., Hanák V., Andreas M., Reiter A., Uhrin M. (2004) **Two new species of bats (Chiroptera) for the fauna of Libya: *Rhinopoma hardwickii* and *Pipistrellus rueppellii***. Myotis, 41–42: 109-124.

Benda P., Hanák V., Červený J. (2011) **Bats (Mammalia: Chiroptera) of the Eastern Mediterranean and Middle East. Part 9. Bats from Transcaucasia and West Turkestan in collection of the National Museum, Prague**. Acta Societatis Zoologicae Bohemicae, 75:159–222.

Benda P., Kiefer A., Hanák V., Veith M. (2004) **Systematic status of African populations of long-eared bats, genus *Plecotus* (Mammalia: Chiroptera)**. Folia Zoologica, 53(1):1–48.

Benda P., Lučan R. K., Shohdi W. M., Porteš M., Horáček I. (2014) **Microbats of the Western Oases of Egypt, Libyan Desert**. Vespertilio, 17:45–58.

Benda P., Lučan R.K., Obuch J., Reiter A., Andreas M., Bačkor P., Bohnenstengel T., Eid E.K., Ševčík M., Vallo P., et al. (2010) **Bats (Mammalia: Chiroptera) of the Eastern Mediterranean and Middle East. Part 8. Bats of Jordan: fauna, ecology, echolocation, ectoparasites**. Acta Societas Zoologicae Bohemicae, 74:185–353.

Benda P., Reiter A., Al-Jumaily M., Nasher A.-K., Hulva P. (2009) **A new species of mouse-tailed bat (Chiroptera: Rhinopomatidae: *Rhinopoma*) from Yemen**. Journal of the National Museum (Prague), Natural History Series, 177 (6):53–68.

Benda P., Ruedi M. (2004) **New data on the distribution of bats (Chiroptera) in Morocco**. Lynx, n. s., 35:13-44.

Benda P., Spitzenberger F., Hanák V., Andreas M., Reiter A., Ševčík M., Šmíd J., Uhrin M. (2014) **Bats (Mammalia: Chiroptera) of the Eastern Mediterranean and Middle East. Part 11. On the bat fauna of Libya II**. Acta Societatis Zoologicae Bohemicae, 78:1–162.

Benda P., Vallo P. (2012) **New look on the geographical variation in *Rhinolophus clivosus* with description of a new horseshoe bat species from Cyrenaica, Libya**. Vespertilio, 16: 69–96.

Benda P., Vallo P., Hulva P., Horáček I. (2012) **The Egyptian fruit bat *Rousettus aegyptiacus* (Chiroptera: Pteropodidae) in the Palaearctic: Geographical variation and taxonomic status**. Biologia, 67(6):1230–1244.

Benda P., Vallo P., Reiter A. (2011) **Taxonomic revision of the genus *Asellia* (Chiroptera: Hipposideridae) with a description of a new species from Southern Arabia**. Acta Chiropterol, 13(2):245–270.

Benda P., Červený J., Konečný A., Reiter A., Ševčík M., Uhrin M., Vallo P. (2010) **Some new records of bats from Morocco (Chiroptera)**. Lynx, 41:151-166.

Bergmans W. (1994) **Taxonomy and biogeography of African fruit bats (Mammalia, Megachiroptera). 4. The genus *Rousettus* Gray, 1821**. Beaufortia, 44:79-126.

Bonhote J.L. (1909) **On a Small Collection of Mammals from Egypt**. Proceedings of the Zoological Society of London, 79(4)788-802.

Churcher C.S. (1991) **The Egyptian fruit bat *Rousettus aegyptiacus* in Dakhleh Oasis, Western Desert of Egypt**. Mammalia, 551:139-143.

DeBlase A. (1972) ***Rhinolophus euryale* and *R. mehelyi* (Chiroptera: Rhinolophidae) in Egypt and Southwest Asia**. Israel Journal of Zoology, 21(1):1–12.

Dietz C. (2005): **Bats Final Report - Operation Wallacea. Sinai 2005**. 23 pp.

Dornburg A., Colosi J.G., Maser C., Reese1 A.T., Watkins-Colwell G.J. (2011) **A survey of the Yale Peabody museum collection of Egyptian mammals collected during construction of the Aswan high dam, with an emphasis on material from the 1962–1965 Yale University prehistoric expedition to Nubia**. Bulletin of the Peabody Museum of Natural History, 52(2):255-272.

Flower S. S. (1932) **Notes on the recent mammals of Egypt, with a list of the species recorded from that kingdom**. Proceedings of the Zoological Society of London 1932, 369-450.

Foui E. (2011) **Operation Wallacea: Sinai bats report.** Available at: https://opwall.com/wp-content/uploads/OpWall_Sinai-bat-report_2011.pdf

Freeman P.W. (1981) **A multivariate study of the family Molossidae (Mammalia, Chiroptera): morphology, ecology, evolution**. Fieldiana Zoology, New Series No. 7, Field Museum of Natural History, Chicago, 173 pp.

Gaisler J., Madkour G. & Pelikan J. (1972) **On the bats of Egypt**. Acta Scientiarum Naturalium Academiae Scientarium Bohemoslovaceae Brno, 6(8):1-40.

Harrison D.L. (1961) **On Savi's Pipistrelle, (*Pipistrellus savii* Bonaparte, 1837) in the Middle East, and a second record of *Nycticeius schlieffeni* Peters 1859 from Egypt**. Senckenbergiana Biologica, 42 (1/2): 41-44.

Harrison D.L. (1964) **The Mammals of Arabia. Vol 1. Introduction, Insectivora, Chiroptera, Primates**. pp 192. Benn, London, 1st edition.

Harrison D.L., Bates P.J.J. (1991) **The mammals of Arabia**. 2nd edition. Harrison Zoological Museum Publication.

Harrison D.L., Makin D. (1988) **Significant new records of Vespertiiionid Bats (Chiroptera: Vespertilionidae) from Israel**. Mammalia, 52(4):593–596.

Hayman R.W. (1948) **The Armstrong College Zoological Expedition to Siwa Oasis (Libyan Desert) 1935: Mammalia**. Proceedings of the Egyptian Academy of Sciences, 4:38–42.

Hill J.E. & Harrison D.L. (1987) **The baculum in the Vespertilioninae (Chiroptera: Vespertilionidae) with a systematic review, a synopsis of *Pipistrellus* and *Eptesicus*, and the descriptions of a new genus and subgenus**. Bulletin of the British Museum (Natural History) Zoology, 52:225-305.

Hoogstraal H. & Traub R. (1963) **The fleas (Siphonaptera) of Egypt. Host-parasite relationships of Indsectivora and Chiroptera**. Journal of the Egyptian Public Health Association, 38(3):111-130.

Hoogstraal H. (1962) **A brief review of the contemporary land mammals of Egypt (including Sinai). 1. Insectivora and Chiroptera**. Journal of the Egyptian Public Health Association, 37(4):143-162.

Kiefer A. (2007) **Phylogeny of Western Palaearctic long-eared bats (Mammalia, Chiroptera, Plecotus): a molecular perspective**. PhD thesis, Johannes Gutenberg-Universität in Mainz.

Kock D. (1969) **Die Fledermaus-Fauna des Sudan**. Abhandlungen der Senckenbergischen Naturforschenden Gesellschaft, Band 521:1:238.

Kock D., Al-Jumaily M. & Nasher A.K. (2002) **Horseshoe bats, genus Rhinolophus Lacépède, 1799 (Mammalia: Chiroptera: Rhinolophidae), of Yemen**. Fauna of Arabia, 19:507–515.

Koopman K.F. (1975) **Bats of the Sudan**. Bulletin of the American Museum of Natural History, 154(4).

Lučan R.K., Šálek M. (2013) **Observation of successful mobbing of an insectivorous bat, *Taphozous nudiventris* (Emballonuridae), on an avian predator, *Tyto alba* (Tytonidae)**. Mammalia, 77(2):235-236.

Macy R.W., Heyneman D., Kuntz R.E. (1961) **Records of trematodes of the families Lecithodendriidae, Dicrocoeliidae, and Heterophyidae from Chiroptera collected in Egypt and Yemen, S. W. Arabia**. Proc. Helminthol. Soc. Washington, 28:13-17.

Madkour G. (1977) **Further observations on bats (Chiroptera) of Egypt**. Agricultural Research Review, 55(1):173–184.

Madkour G. (1977) ***Rousettus aegyptiacus* (Megachiroptera) as a fruit eating bat in A.R. EGYPT**. Agricultural research review, 55(1):167-172.

Madkour G. (1978) **Significance of the distal part of the humerus in the identification of Egyptian bats**. Zoologischer Anzeiger, 201(5/6):387-390.

Nader I.A. & Kock D. (1983) **Notes on some bats from the Near East (Mammalia: Chiroptera)**. Zeitschrift für Säugetierkunde, 48:1–9.

Nader I.A. (1982) **New distributional records of bats from the Kingdom of Saudi Arabia (Mammalia: Chiroptera)**. Journal of Zoology, 198(1):69-82.

Osborn D.J. (1988) **New bat records from the Red Sea Mountains of Egypt**. Mammalia, 52(4):596-598.

Owen R.D., Qumsiyeh M.B. (1987) **The subspecies problem in the Trident leaf-nosed bat, *Asellia tridens*: homomorphism in widely separated populations**. Zeitschrift Für Saeugetierkunde, 6:329-337.

Qumsiyeh M.B. (1985) **The bats of Egypt**. Special Publication of the Texas Technological University, 23: 1-102.

Qumsiyeh M.B. (1996) **Mammals of the Holy Land**. Texas Tech University Press, Lubbock.

Robbins L.W., Sarich V.M. (1988) **Evolutionary relationships in the family Emballonuridae (Chiroptera)**. Journal of Mammalogy, 69(1):1-13.

Sanborn C.C. & Hoogstraal H. (1955) **The identification of Egyptian bats**. Journal of the Egyptian Public Health Association, 30(4):103-119.

Saoud M.F. & Ramadan M.M. (1977): **On a new trematode, *Prohemistomum azimi* n. sp. (Trematoda: Cyathocotylidae) from the Egyptian slit-faced bat**. Zeitschrift für Parasitenkunde, 53(3):281–285.

Schwann H. (1905) **A list of the mammals collected by the Hon. N. C. Rothschild, the Hon. F. R. Henley, and Mr. A. F. R. Wollaston in Egypt and the Soudan in January, February, and March 1904**. Novitates Zoologicae, 12:1–5.

Setzer H.W. (1952) **Notes on mammals from the Nile Delta region of Egypt**. Proceedings of the United States National Museum, 102(3305):343–369.

Shehab A., Mamkhair I., Amr Z. (2006) **First record of the Lesser Horseshoe bat, *Rhinolophus hipposideros* (Bechstein, 1800) (Rhinolophidae, Chiroptera) from Syria**. Hystrix, the Italian Journal of Mammalogy, 17(2):161-166.

Sohail S, Sameeh A.M. & Kareem M.S. (2014) **Population studies on two sympatric bat species: *Taphozous Perforatus* and *Rhinopoma Hardwickii* (Chiroptera: Microchiroptera) from Egypt**. Egyptian Journal of Zoology, 64:13–31.

Soliman S. & Mohallal E. (2014) **A survey of the mammalian fauna of Siwa Oasis, Egypt**. Egyptian Journal of Zoology, 61:171–186.

Van Cakenberghe V., De Vree F. (1998) **Systematics of African *Nycteris* (Mammalia: Chiroptera) Part III. The *Nyteris thebaica* group**. Bonner Zoologische Beitrage, 48(2):123-166.

Wassif K. & Hoogstraal H. (1953) **The Mammals of South Sinai, Egypt**. Proceedings of the Egyptian Academy of Science, 9:63–79.

Wassif K. (1954) **On a collection of mammals from northern Sinai**. Bulletin de l'Institute du Désert de l'Egypte, 3(1):107-118.

Wassif K., Madkour G. & Soliman S. (1984) **Fauna and Flora of Egypt. 1. On a collection of bats from Egypt**. Academy of Scientific Research and Technology, Natural History Museum of Egypt, 2:1-36.

Yassen A.E., Hassan H.A., Kawashti L.S. (1994) **Comparative study of the karyotypes of two Egyptian species of bats, *Taphozous perforatus* and *Taphozous nudiventris* (Chiroptera: Mammalia)**. Experientia, 50(11):111-1114.

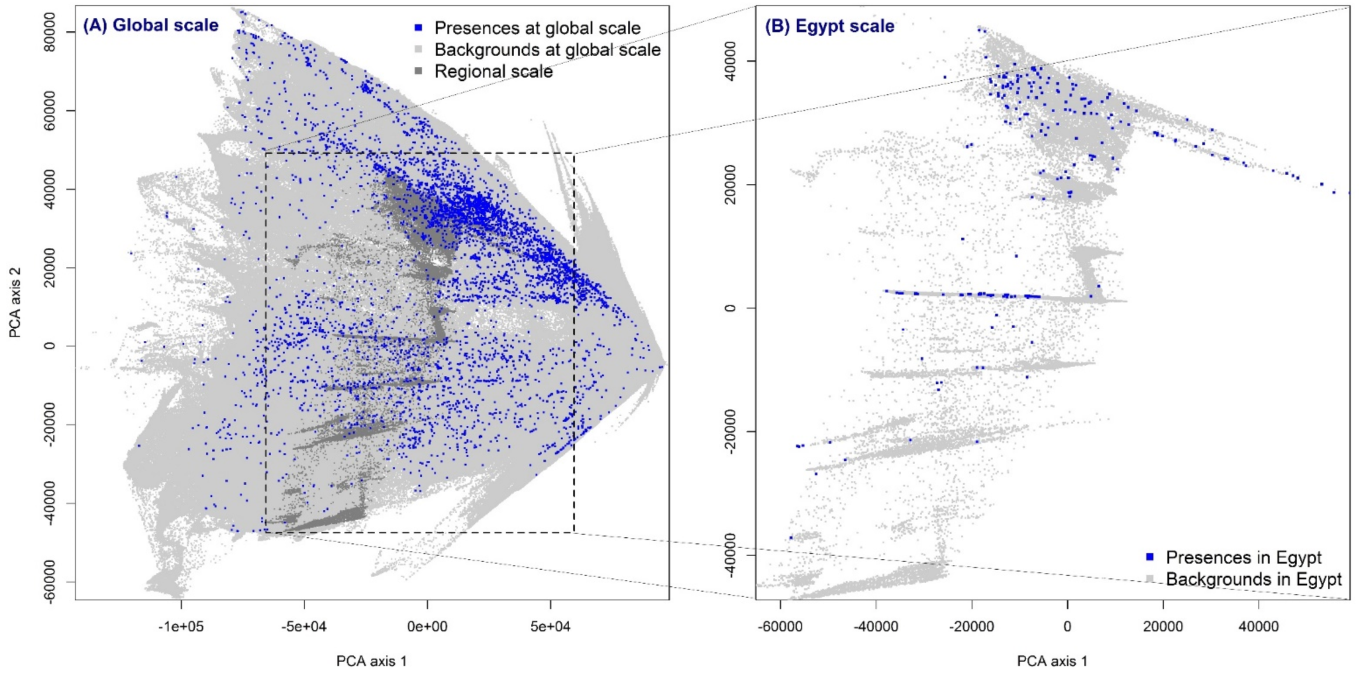

Fig. S1: **Left:** scatterplot of the first two PCA axes of all available environmental covariates within the entire study area. The first two axes account for 94.2% of the environmental variation (76.4% and 17.8%, respectively). **Right:** A close-up view of the available environmental space in Egypt (highlighted in dashed rectangle in the left plot). Blue dots represent presence locations for any study species; light grey points represent pixels without any sightings; dark grey points represent the available environmental space in Egypt.

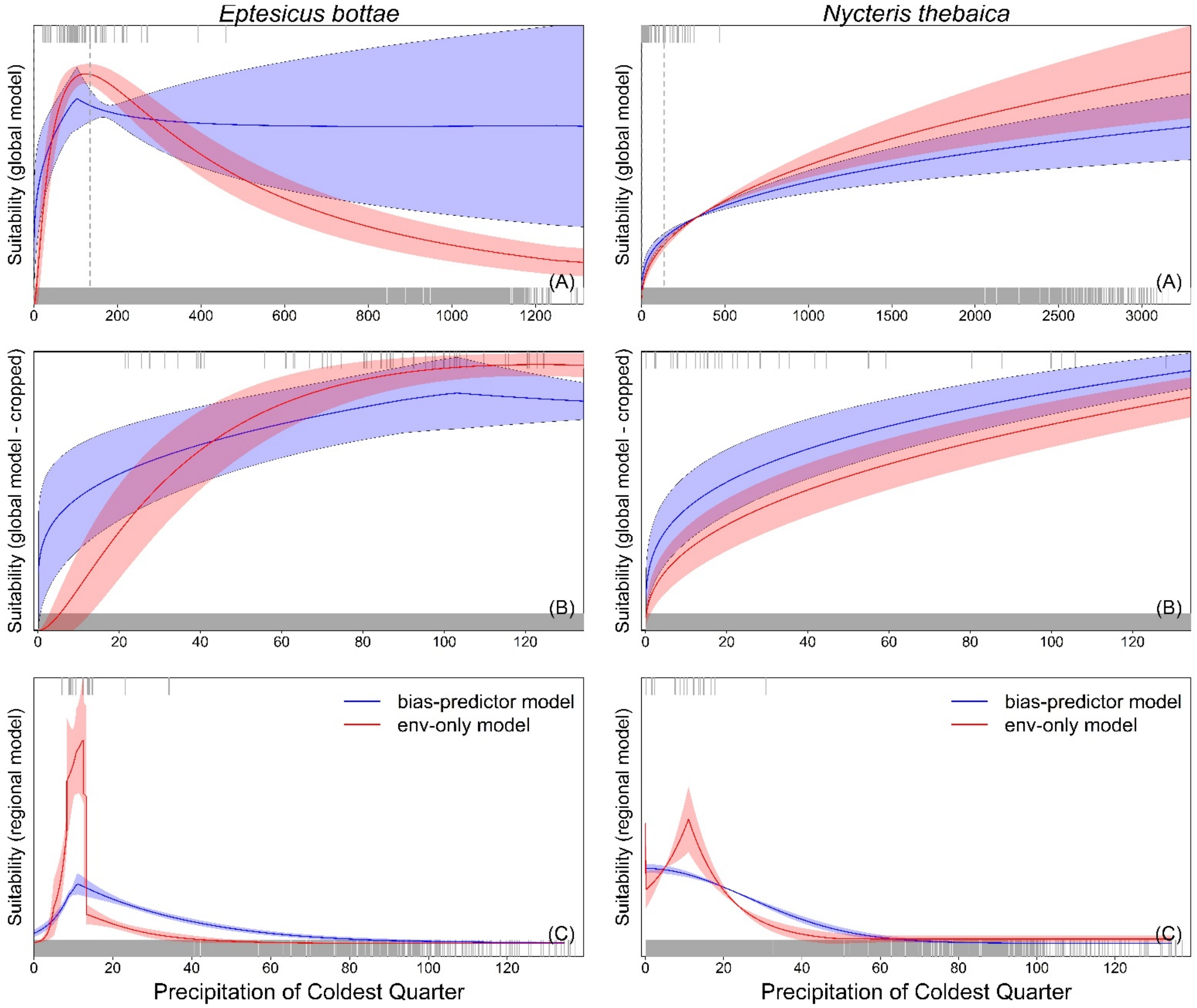

Fig. S2: Maxent's response to precipitation of coldest quarter for *Eptesicus bottae* (left) and *Nycteris thebaica* (right) in **(A)** the global model, **(B)** the global model cropped to the precipitation range available in Egypt, and **(C)** the regional model within Egypt. In each plot, the lower rug indicates precipitation values at background locations while the upper rug indicates precipitation values at presence locations. Red and blue lines represent environment-only and Bias-predictor models, respectively. Lines and their shaded areas represent the mean and standard deviation of species response. Note that the predicted values of global (A) or regional (C) models were scaled to sum to one for comparison consistency.

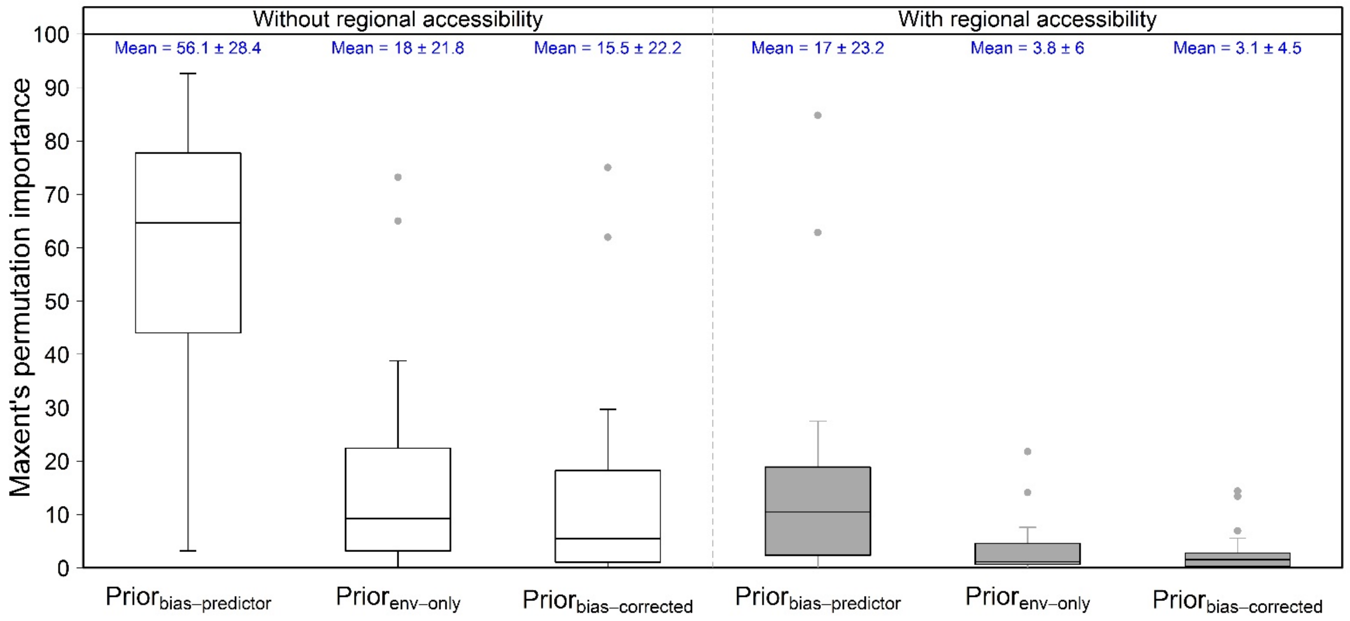

Fig. S3: Boxplots of mean permutation importance (%) of the prior variables, without (left) versus with (right) regional bias variables incorporation, as reported in Maxent.

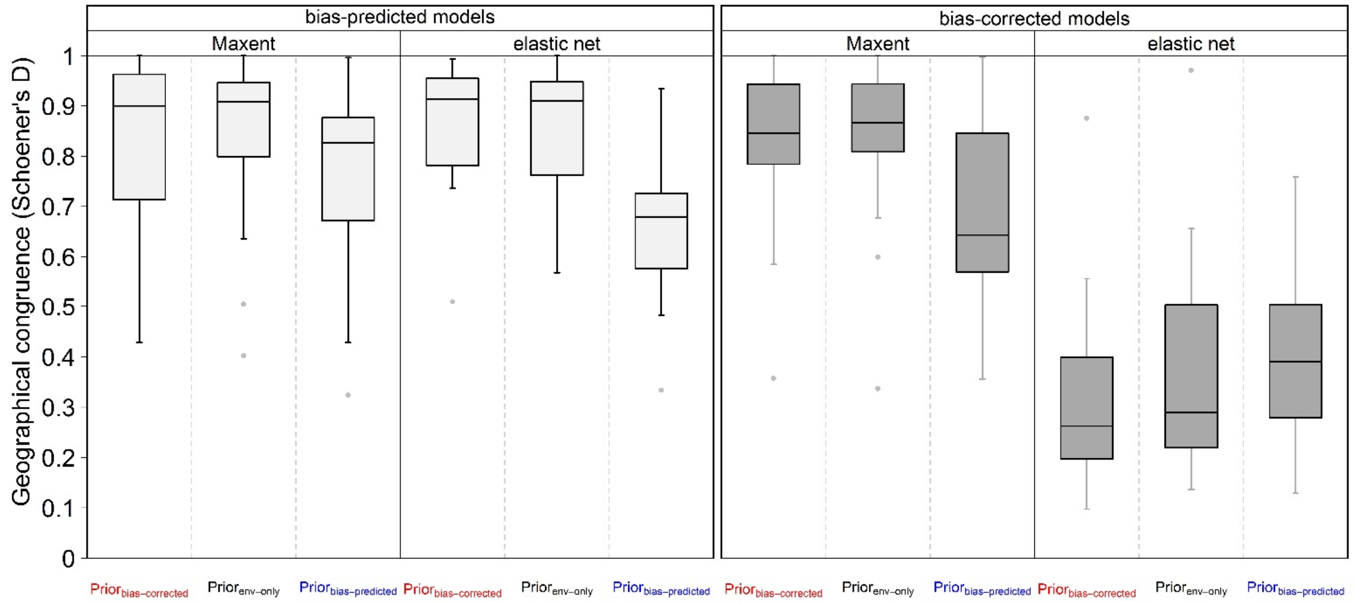

Fig. S4: Geographical congruence between the predictions of regional SDMs calibrated without priors and the three versions of regional models that used a prior variable. Here (in contrast to Fig. 5), bias variables were incorporated as predictors in the regional SDMs.

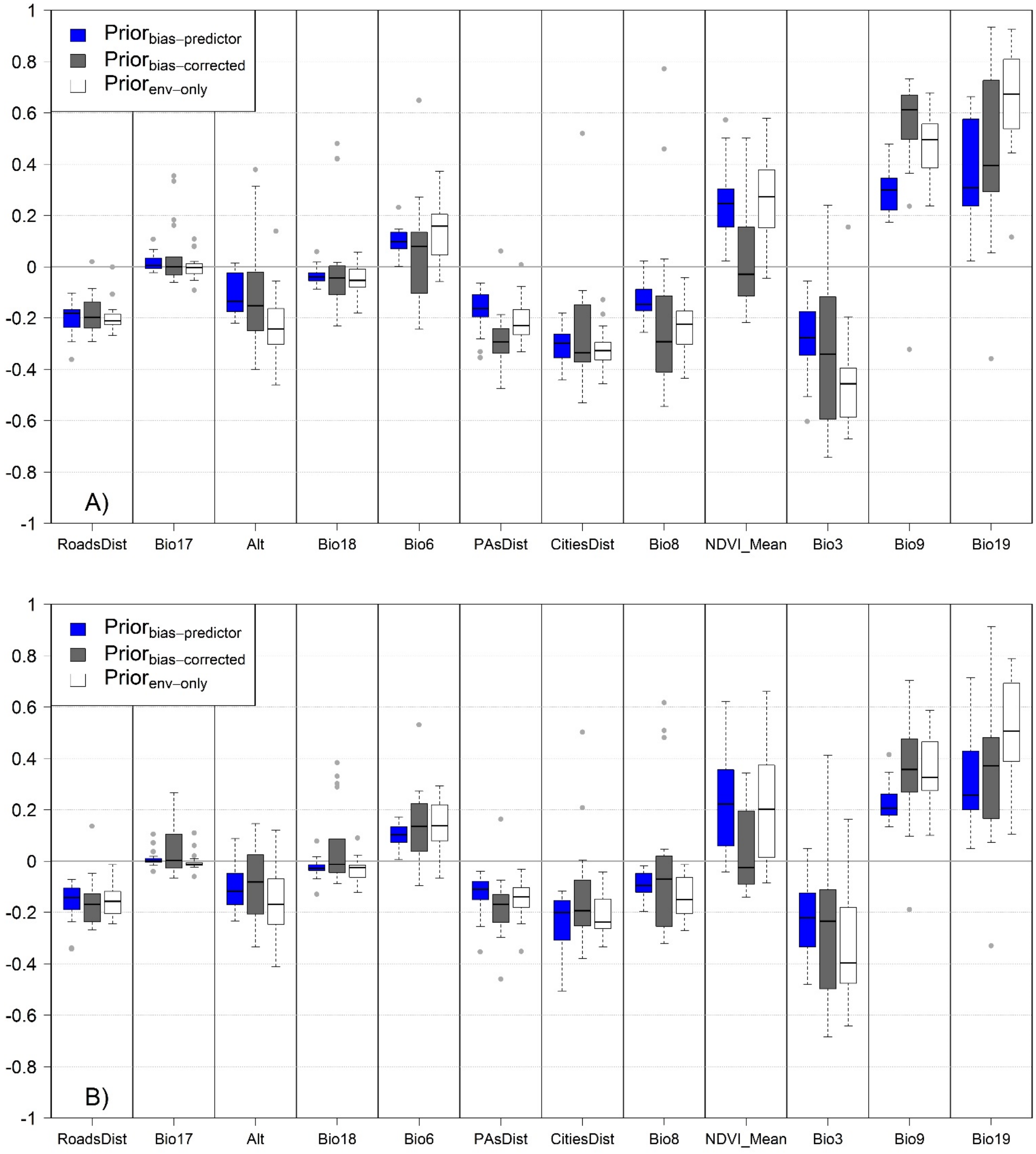

Fig. S5: Boxplots for the Pearson's correlation between the three priors and environmental variables used to run regional models, for (A) Maxent and (B) elastic net.

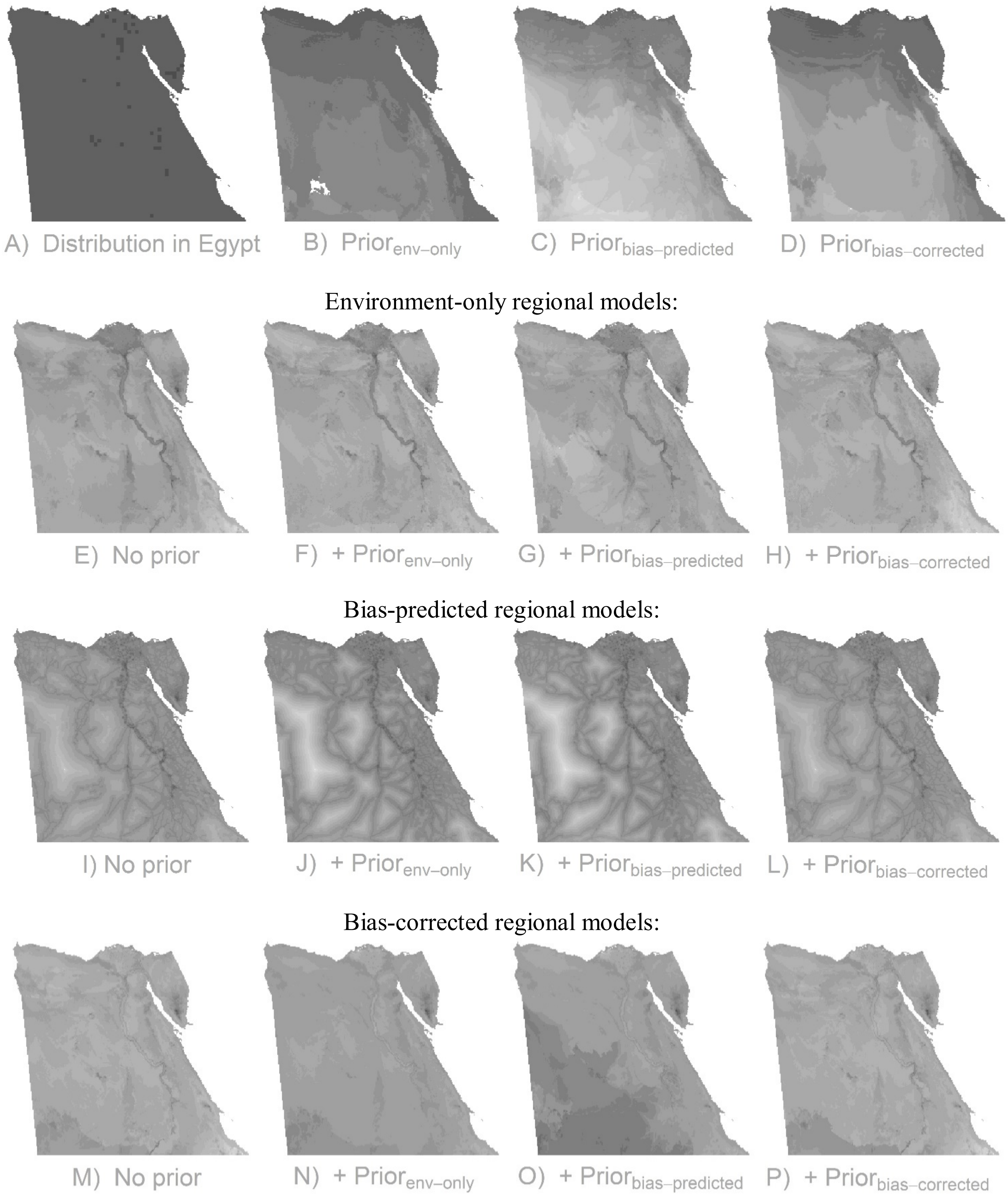

Fig. S6: Example maps

- **A:** presence locations of the Egyptian fruit bat (*Rousettus aegyptiacus*) in Egypt, used to calibrate regional SDMs in Egypt.
- **B, C, D:** predictions from the global models in Egypt [priors].
- **E, I, M:** predictions from regional models in Egypt, with no priors incorporated into the models. **E:** prediction from environment-only regional model; **I**: prediction from bias-predicted regional models; **M**: prediction from bias-corrected regional models.
- **F, G, H:** similar to **E**, with priors (**B, C, D**) incorporated to the model, respectively.
- **J, K, L:** similar to **I**, with priors (**B, C, D**) incorporated to the model, respectively
- **M, N, O:** similar to **M**, with priors (**B, C, D**) incorporated to the model, respectively.

In all maps, the darker the colour, the higher the relative suitability (log-scaled).

Table S1: List of species used or excluded in this study

A. selected species

Asellia tridens (trident leaf-nosed bat)
Eptesicus bottae (Botta's serotine bat)
Hypsugo ariel (fairy pipistrelle)
Nycteris thebaica (Egyptian slit-faced bat)
Otonycteris hemprichii (Hemprich's long-eared bat)
Pipistrellus kuhlii (Kuhl's pipistrelle)
Pipistrellus rueppellii (Rueppell's pipistrelle)
Plecotus christii (desert long-eared bat)
Rhinolophus clivosus (Arabian horseshoe bat)
Rhinolophus mehelyi (Mehely's horseshoe bat)
Rhinopoma cystops (lesser mouse-tailed bat)
Rhinopoma microphyllum (greater mouse-tailed bat)
Rousettus aegyptiacus (Egyptian fruit bat)
Tadarida aegyptiaca (Egyptian free-tailed bat)
Tadarida teniotis (European free-tailed bat)
Taphozous nudiventris (naked-bellied tomb bat)
Taphozous perforatus (tomb bat)

B. excluded species

Barbastella leucomelas (Sinai barbastelle)
Nycticeinops schlieffeni (Schlieffen's bat)
Pipistrellus deserti (desert pipistrelle)
Rhinolophus hipposideros (lesser horseshoe bat)

Table S2: List of environmental predictors used either at global or regional scale

Variable	Global scale	Regional scale
Altitude	YES	YES
**Bio1**: Annual mean temperature		
**Bio2**: Mean diurnal range (mean of monthly (max temp - min temp))	YES	
**Bio3**: Isothermality (Bio2/Bio7) (* 100)		YES
**Bio4**: Temperature seasonality (standard deviation *100)	YES	
**Bio5**: Maximum temperature of warmest month		
**Bio6**: Minimum temperature of coldest month		YES
**Bio7**: Temperature annual range (Bio5-Bio6)		
**Bio8**: Mean temperature of wettest quarter	YES	YES
**Bio9**: Mean temperature of driest quarter	YES	YES
**Bio10**: Mean temperature of warmest quarter		
**Bio11**: Mean temperature of coldest quarter		
**Bio12**: Annual precipitation		
**Bio13**: Precipitation of wettest month		
**Bio14**: Precipitation of driest month	YES	
**Bio15**: Precipitation seasonality (coefficient of variation)	YES	
**Bio16**: Precipitation of wettest quarter		
**Bio17**: Precipitation of driest quarter		YES
**Bio18**: Precipitation of warmest quarter		YES
**Bio19**:Precipitation of coldest quarter	YES	YES
Potential evapotranspiration		
Actual evapotranspiration		
Aridity index		
Soil-Water balance		
Minimum NDVI	YES	
Standard deviation of NDVI	YES	
